# Modulation of the stability and activities of HIV-1 Tat by its ubiquitination and carboxyl-terminal region

**DOI:** 10.1186/2045-3701-4-61

**Published:** 2014-10-07

**Authors:** Linlin Zhang, Juan Qin, Yuanyuan Li, Jian Wang, Qianqian He, Jun Zhou, Min Liu, Dengwen Li

**Affiliations:** State Key Laboratory of Medicinal Chemical Biology, College of Life Sciences, Nankai University, Tianjin, 300071 China

**Keywords:** Tat, Ubiquitination, Carboxyl-terminal region, Stability, Activity

## Abstract

**Background:**

The transactivator of transcription (Tat) protein of human immunodeficiency virus type 1 (HIV-1) is known to undergo ubiquitination. However, the roles of ubiquitination in regulating Tat stability and activities are unclear. In addition, although the 72- and 86-residue forms are commonly used for *in vitro* studies, the 101-residue form is predominant in the clinical isolates of HIV-1. The influence of the carboxyl-terminal region of Tat on its functions remains unclear.

**Results:**

In this study, we find that Tat undergoes lysine 48-linked ubiquitination and is targeted to proteasome-dependent degradation. Expression of various ubiquitin mutants modulates Tat activities, including the transactivation of transcription, induction of apoptosis, interaction with tubulin, and stabilization of microtubules. Moreover, the 72-, 86- and 101-residue forms of Tat also exhibit different stability and aforementioned activities.

**Conclusions:**

Our findings demonstrate that the ubiquitination and carboxyl-terminal region of Tat are critical determinants of its stability and activities.

## Background

The HIV-1 transactivator of transcription Tat undergoes multiple posttranslational modifications, including acetylation, methylation, and ubiquitination, which regulate Tat-mediated transactivation of HIV long terminal repeat (LTR) [1–8]. Tat acetylation has been well characterized to be fine-tuned by histone acetyltransferases (HATs) and histone deacetylases (HDACs) at specific lysine residues and is involved in the regulation of Tat activities [1, 4, 9–14]. The mutation of certain lysine residues in Tat significantly affects Tat activities such as transactivation of transcription and induction of apoptosis [15]. Given that ubiquitination is another posttranslational modification of lysine residues, it is reasonable to speculate that the influences on Tat activities caused by lysine mutation may be partially attributed to altered ubiquitination of Tat.

In support of this speculation, Tat has been reported to be subjected to ubiquitination [2]. According to the previous study, Tat undergoes lysine 63-linked ubiquitination mediated by the proto-oncoprotein Hdm2, which further regulates Tat transactivation activity through a non-proteolytic pathway [2]. Modification of Tat by lysine 63-linked polyubiquitin chains does not affect Tat stability [2]. Whether the stability of Tat is modulated by the ubiquitin-proteasome system, either through the canonical lysine 48-linked ubiquitination or the non-canonical signals such as lysine 29-linked ubiquitination [16], remains to be determined. Besides, the roles of ubiquitination in the regulation of the diverse functions of Tat are largely unknown.

The full-length 101-residue form of Tat (hereafter, Tat101 or just Tat) is encoded by HIV-1 *tat* gene composed of two exons. The first exon encodes a truncated form of Tat with only the first 72 amino acids (hereafter Tat72). It is generated in the late stage of HIV-1 infection cycle. The 86-residue truncated form of Tat (hereafter Tat86), produced early in HIV-1 infection, is generated due to a premature stop codon within the second exon. Though the full-length form of Tat is predominant in HIV-1 clinical isolates [17], Tat86 and Tat72 are more widely used for *in vitro* studies, leaving the carboxyl-terminal region unconsidered.

Nevertheless, several studies have witnessed the significance of the carboxyl-terminal region of Tat for its activities [18–21]. For instance, the second exon of *tat* gene has been demonstrated to have an important function for *in vivo* replication [18]. Additionally, Tat101 and Tat86 have been reported to be distinct from each other in the abilities of transactivation of HIV-1 LTR and induction of apoptosis of T cells [19]. However, a detailed and systematic comparison of the activities of the full-length Tat and two truncated forms is still needed to fully decipher the biological importance of the carboxyl-terminal region of Tat in the regulation of its functions.

In this study, we provide the first evidence that Tat undergoes lysine 48-linked ubiquitination and is targeted to proteasome-dependent degradation. Expression of various ubiquitin mutants modulates diverse activities of Tat. In addition, we find that the stability and activities of the 72-, 86- and 101-residue forms of Tat are distinct. Our results suggest that the ubiquitination and carboxyl-terminal region of Tat are involved in the regulation of Tat stability and activities.

## Results

### The ubiquitination and carboxyl-terminal region of Tat regulate the stability of Tat

**Figure 1:**
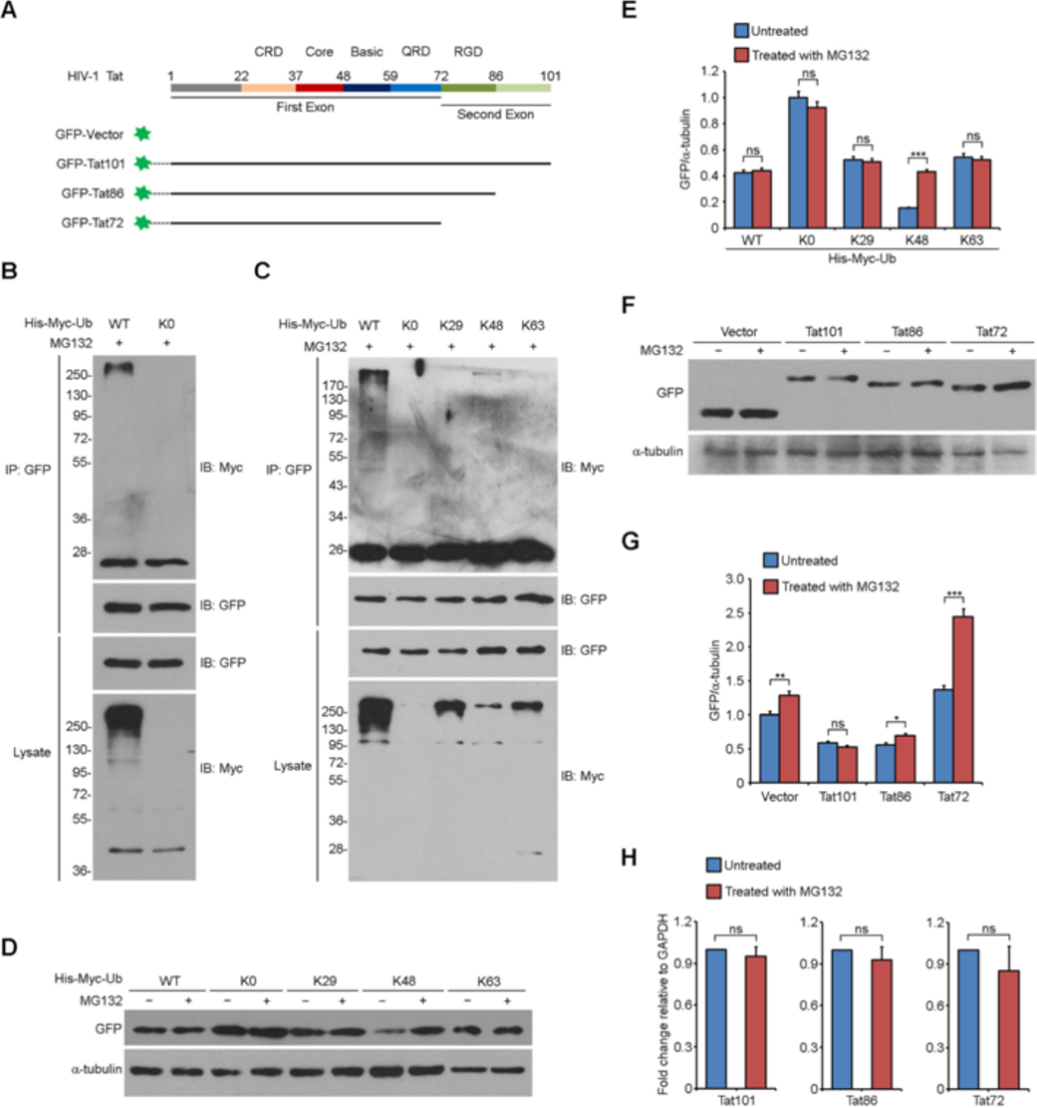
**The ubiquitination and carboxyl-terminal region of Tat regulate the stability of Tat. (A)** Schematic representation and functional domains of HIV-1 Tat and schematic diagrams of GFP-tagged Tat101 and two truncated mutants. CRD, cysteine-rich domain; Core, conserved core region; Basic, region of basic amino acids; QRD, glutamine-rich domain; RGD, region of Arg-Gly-Asp sequence. **(B and** **C)** 293 T cells were cotransfected with GFP-Tat101 and His-Myc-tagged WT ubiquitin or the lysine mutants (K0, K29, K48, and K63) and treated with MG132. Anti-GFP immunoprecipitates and cell lysates were immunoblotted with anti-Myc or anti-GFP antibodies. **(D)** 293 T cells were cotransfected with GFP-Tat101 and His-Myc-tagged WT ubiquitin or the lysine mutants and treated with (+) or without (-) MG132. Cell lysates were subjected to immunoblot analysis with antibodies against GFP or α-tubulin. Anti-α-tubulin western blot was done as a loading control. **(E)** Quantification of the results in **(D)**. Bars represent the relative ratios of GFP over α-tubulin levels normalized to the untreated ubiquitin-K0 mutant transfection group. **(F)** 293 T cells were transfected with GFP-Tat101, GFP-Tat86, GFP-Tat72 or GFP alone and treated with (+) or without (-) MG132. Cell lysates were immunoblotted with anti-GFP or anti-α-tubulin antibodies. **(G)** Quantification of the results in **(F)**. Bars represent the relative ratios of GFP over α-tubulin levels normalized to the untreated GFP vector transfection group. **(H)** Quantitative real-time PCR analysis of gene expression in 293 T cells transfected with GFP-Tat101, GFP-Tat86, or GFP-Tat72 and treated with (+) or without (-) MG132. GAPDH was used for normalization. Two-tailed Student’s *t*-test for all graphs. *P < 0.05, **P <0.01, ***P < 0.001; ns, not significant. Mean and standard deviations were derived from three independent biological replicates. Cropped blots are used in this figure, and the gels were run under the same experimental conditions.

To investigate the roles of the ubiquitination and carboxyl-terminal region of Tat in the regulation of its stability, we firstly constructed GFP-tagged full-length Tat (GFP-Tat101) and the truncated forms (GFP-Tat86 and GFP-Tat72) as shown in Figure 1A. By immunoprecipitation assays, we confirmed that Tat was subjected to ubiquitination when cotransfected with His-Myc-tagged wild-type (WT) ubiquitin but not the ubiquitin-K0 mutant (Figure 1B). To further determine the type of polyubiquitin linkage with which Tat is modified, we constructed ubiquitin-K29, -K48, and -K63 mutants which contain only a single lysine (K29, K48, and K63, respectively) with all the other lysines mutated to arginine. As shown in Figure 1C, ubiquitination of Tat could only be detected in ubiquitin-K48 mutant cotransfection group, but was much weaker as compared to ubiquitin-WT group. This indicates that other types of Tat ubiquitination may exist though they were not observed by our approach. We still examined the effects of ubiquitin-K29 and -K63 mutants on Tat activities through cotransfection.

To evaluate the effects of Tat ubiquitination on its stability, 293 T cells were cotransfected with GFP-Tat101 and various ubiquitin mutants and treated with or without the proteasome inhibitor MG132 for 8 hours before collection. No substantial effect on Tat levels was observed by MG132 treatment except for the ubiquitin-K48 mutant cotransfection group (Figure 1D and E), which indicated that the lysine 48-linked ubiquitination of Tat targeted it to proteasome-dependent degradation. However, Tat in ubiquitin-WT cotransfection group showed little response to MG132 treatment. This might be due to the fact that lysine 48-linked ubiquitination is not the dominant type of ubiquitination of Tat, which has been indicated by Figure 1C. The ubiquitin-K48 mutant contains only a single lysine with all the other lysines mutated to arginine, while the wild-type ubiquitin contains 7 lysines. The other lysines in wild-type ubiquitin may competitively inhibit lysine 48-linked ubiquitination and proteasome-dependent degradation of Tat (Figure 1D, lane 1 *vs* lane 7).

We next assessed the influence of the carboxyl-terminal region of Tat on its stability. According to our data, MG132 treatment had little effect on the protein level of Tat101, only slightly affected that of Tat86, but increased that of Tat72 by nearly 45% (*P* < 0.0001) (Figure 1F and G). By quantitative real-time PCR, we found that the mRNA levels of Tat101, Tat86, and Tat72 were not significantly changed by MG132 treatment (Figure 1H). This finding suggests that the carboxyl-terminal region truncation of Tat makes it fragile and sensitive to proteasome-dependent degradation. Collectively, these results demonstrate that the stability of Tat is modulated by its ubiquitination and carboxyl-terminal region.

### The ubiquitination or carboxyl-terminal region of Tat has little effect on its subcellular localization

**Figure 2:**
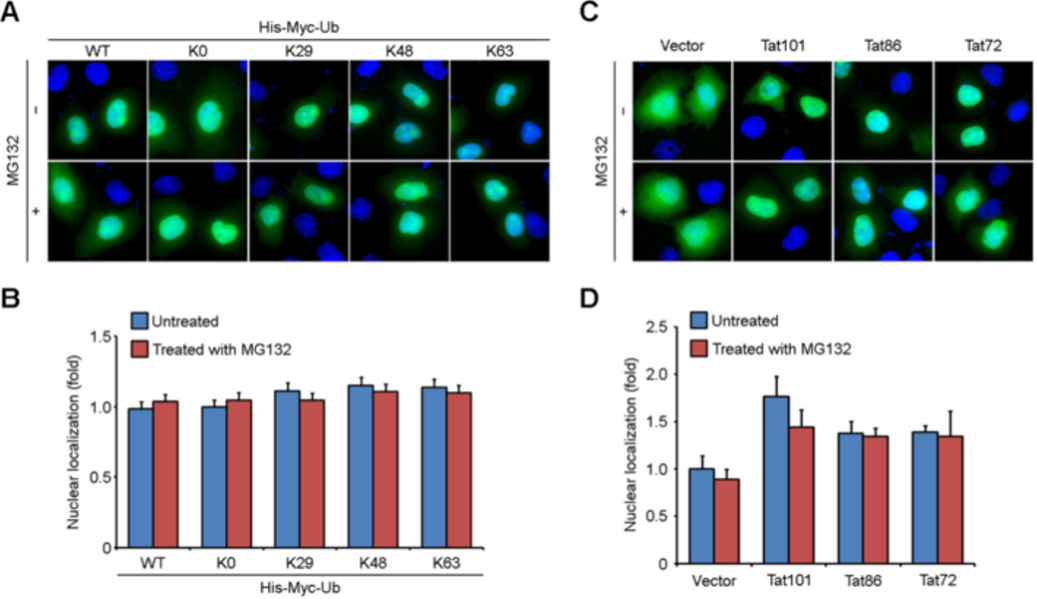
**The ubiquitination or carboxyl-terminal region of Tat has little effect on its subcellular localization. (A)** HeLa cells were cotransfected with GFP-Tat101 and His-Myc-tagged WT ubiquitin or the lysine mutants and treated with (+) or without (-) MG132. Cells were then stained with the DNA dye DAPI. The subcellular localization of GFP-Tat101 was examined by fluorescence microscopy. **(B)** Experiments were performed as in **(A)**, and the percentage of nuclear GFP-Tat101 was quantified via ImageJ software. Bars represent the relative folds normalized to the untreated ubiquitin-K0 mutant transfection group. Experiments were done in duplicate and results were expressed as the mean ± SD. **(C)** HeLa cells were transfected with GFP-Tat101, GFP-Tat86, GFP-Tat72 or GFP alone and treated with (+) or without (-) MG132. Cells were then subjected to nuclear staining with DAPI. The subcellular localization of GFP alone or GFP fusion proteins was analyzed by fluorescence microscopy. **(D)** Experiments were performed as in **(C)**, and the percentage of nuclear GFP or GFP fusion proteins was quantified via ImageJ software. Bars represent the relative folds normalized to the untreated GFP vector transfection group. Experiments were done in duplicate and results were expressed as the mean ± SD.

Given that posttranslational modification may influence protein subcellular localization, and the distribution patterns vary among different variants of certain proteins, we asked whether the ubiquitination or carboxyl-terminal region of Tat affects its distribution. HeLa cells were transfected with the indicated plasmids and examined by fluorescence microscopy. The nuclear localization of Tat was quantified via ImageJ software by measuring the ratio of GFP intensity inside the nucleus over that in the whole cell.No substantial difference in Tat localization was observed when full-length Tat was cotransfected with various ubiquitin mutants in the absence or presence of MG132 (Figure 2A and B). Full-length Tat and the two truncated variants displayed similar distribution patterns with a slight decrease in nuclear localization of the truncated forms, and MG132 caused no remarkable changes (Figure 2C and D). Altogether, these observations suggest that the ubiquitination or carboxyl-terminal region of Tat has little effect on its localization.

### Tat transactivation activity is modulated by its ubiquitination and carboxyl-terminal region

It has been reported that posttranslational modifications of Tat, such as acetylation, methylation and phosphorylation, regulate its transactivation activity [22]. Additionally, previous studies have documented a significant difference in the transactivation activities of full-length Tat and the truncated variant Tat86 [19]. We thus systematically analyzed the effects of the ubiquitination and carboxyl-terminal region of Tat on its transactivation activity.

**Figure 3:**
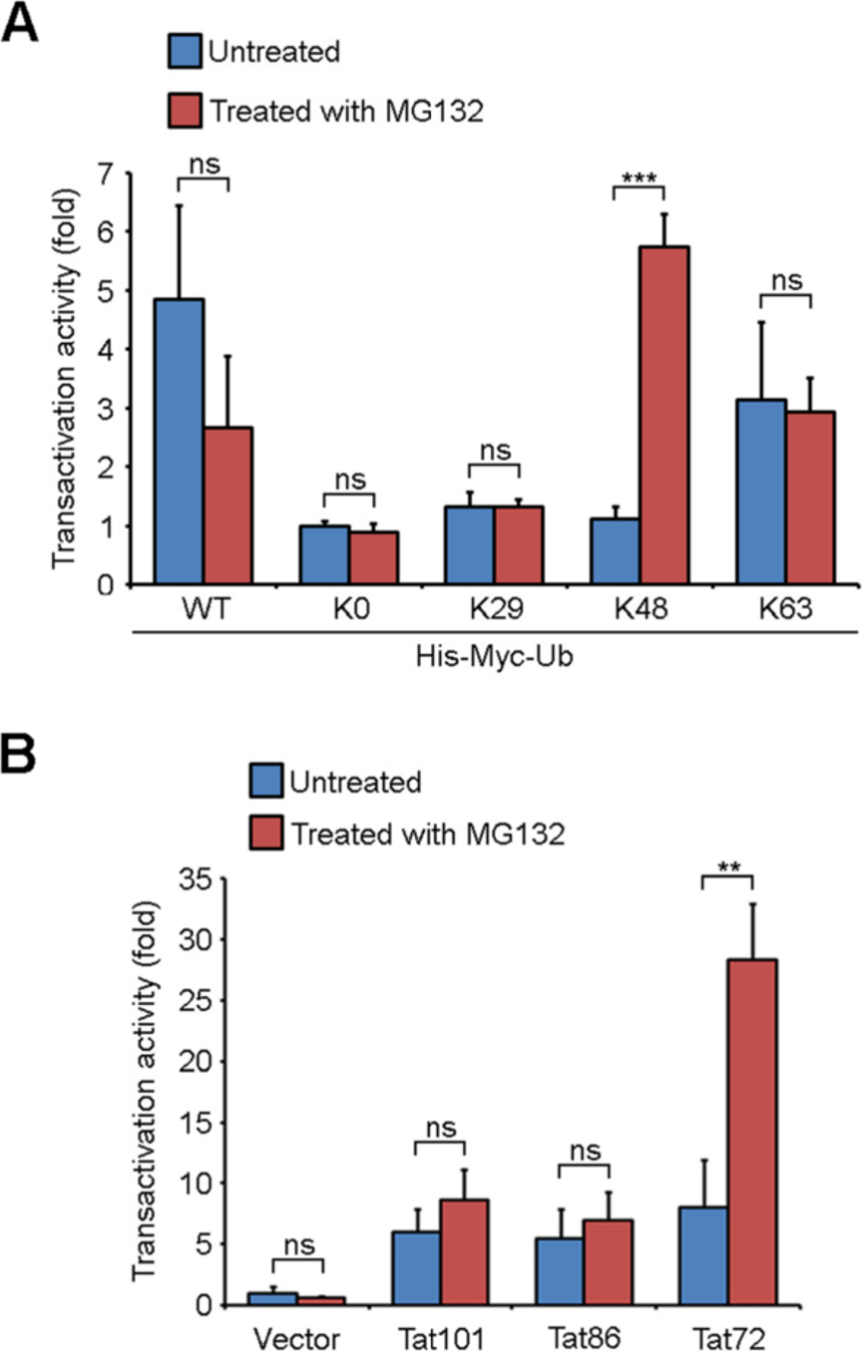
**Tat transactivation activity is modulated by its ubiquitination and carboxyl-terminal region. (A)** 293 T cells were cotransfected with an HIV-1 LTR-driven luciferase plasmid, a GFP-Tat101 plasmid, and various ubiquitin mutants and treated with or without MG132. Luciferase activity was then measured and normalized to GFP intensity. Bars represent the relative folds of transactivation activity obtained from two experiments done in triplicate and normalized against the untreated ubiquitin-K0 mutant transfection group. Results were expressed as the mean ± SD. **(B)** 293 T cells were cotransfected with an HIV-1 LTR-driven luciferase plasmid and plasmids expressing GFP fusion proteins or GFP alone and treated with or without MG132. The relative folds of transactivation activity were obtained as in **(A)**. Results were expressed as the mean ± SD. Two-tailed Student’s *t*-test for all graphs. *P < 0.05, **P <0.01, ***P < 0.001; ns, not significant.

Compared to the untreated ubiquitin-K0 cotransfection group, cotransfection with ubiquitin-WT or -K63 led to a five- or three-fold increase in Tat transactivation activity respectively, while cotransfection with ubiquitin-K29 or -K48 caused only a slight increase (Figure 3A). The addition of MG132 showed no considerable effect on Tat transactivation activity in ubiquitin-K0, -K29 and -K63 cotransfection groups, but brought about opposite effects in ubiquitin-WT and -K48 groups (Figure 3A). However, the results of statistical analysis indicated that the changes of transactivation activity caused by MG132 treatment in ubiquitin-WT cotransfection groups were not significant (*P* = 0.1183).

As for the effects of the carboxyl-terminal region of Tat on its transactivation activity, luciferase reporter assays were performed and the results revealed that full-length Tat and the two truncated mutants resembled each other in transactivation ability in the absence of MG132 (Figure 3B). Intriguingly, MG132 exerted slight effect on the transactivation activity of Tat101 or Tat86, but markedly increased that of Tat72 (*P* = 0.0043) (Figure 3B). These findings together indicate that Tat transactivation activity is regulated by its ubiquitination and carboxyl-terminal region.

### The ability of Tat to induce apoptosis is regulated by its ubiquitination and carboxyl-terminal region

Tat acetylation has been characterized to be involved in the regulation of Tat-mediated apoptosis [15, 23]. Moreover, Tat86 has been reported to trigger slightly more apoptosis in CD4^+^ T cells than the full-length form [19]. We thus examined the influences of Tat ubiquitination on its induction of apoptosis, and further compared the abilities of Tat101 and the two truncated forms to trigger apoptosis.

**Figure 4:**
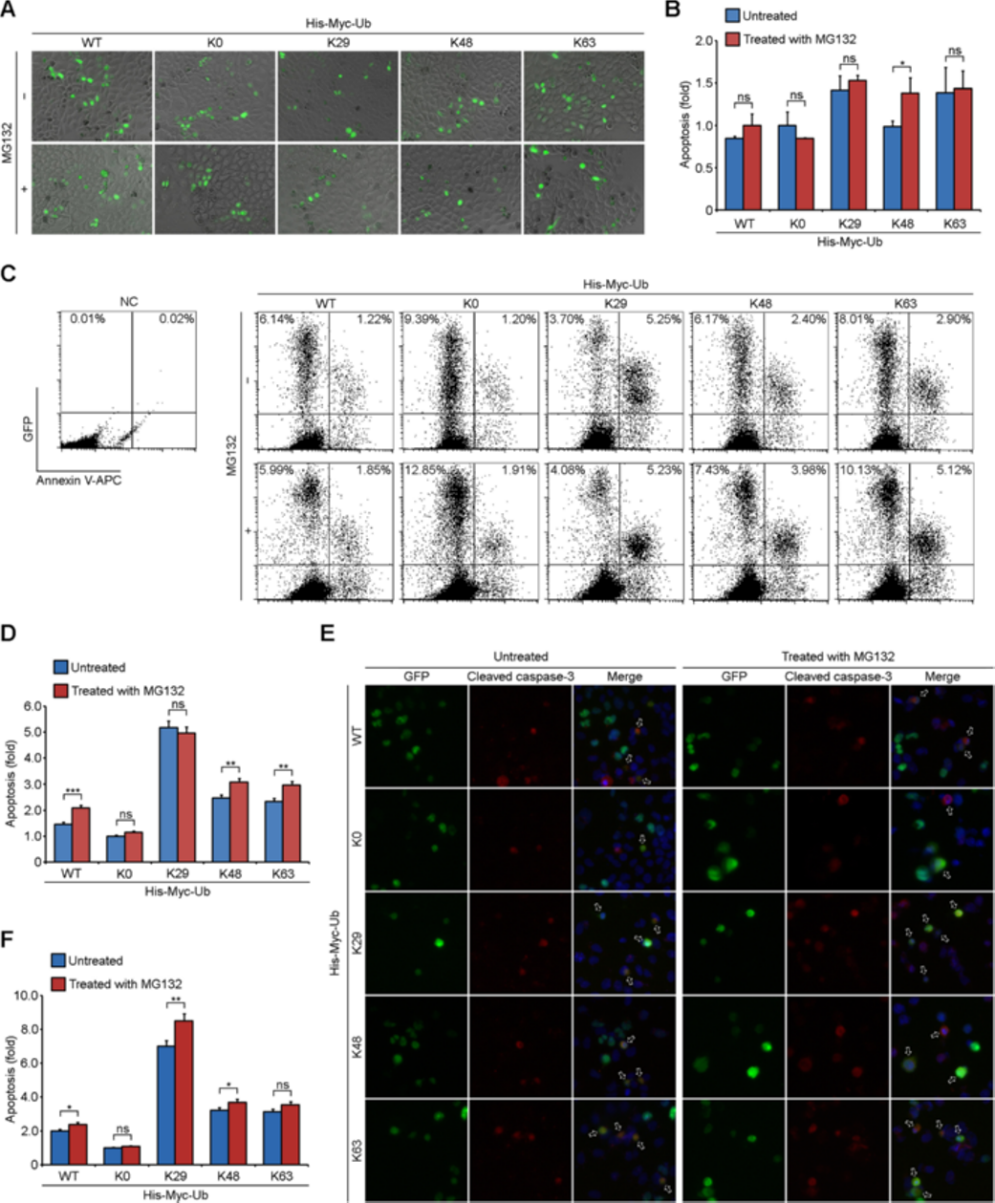
**Tat-induced apoptosis is regulated by its ubiquitination. (A)** HeLa cells were cotransfected with GFP-Tat101 and His-Myc-tagged WT ubiquitin or the lysine mutants. 72 hours post-transfection, cells were treated with (+) or without (-) MG132 and incubated for additional 24 hours. Apoptotic cells were examined via the fluorescence microscope by analyzing cell morphology of GFP-positive cells. **(B)** Quantification of the results in **(A)**. The number of apoptotic cells was counted and the percentage of apoptotic cells was calculated. Bars represent the relative folds of Tat-induced apoptosis normalized to the untreated ubiquitin-K0 mutant transfection group. Experiments were done in duplicate, six fields per group were counted, and the results were expressed as the mean ± SD. **(C)** HeLa cells were transfected and treated as in **(A)**, and the apoptotic cells were examined by Annexin V-APC staining coupled with flow cytometry. Mock transfected HeLa cells without Annexin V-APC staining were used as negative control (NC). **(D)** Quantification of the results in **(C)**. Bars represent the relative folds of Tat-induced apoptosis normalized to the untreated ubiquitin-K0 mutant transfection group. Mean and standard deviations were derived from two independent experiments done in duplicate. **(E)** HeLa cells were transfected and treated as in **(A)**, and stained with anti-cleaved caspase-3 antibodies and the DNA dye DAPI. Apoptotic cells were indicated by hollow arrows. **(F)** Quantification of the results in **(E)**. Bars represent the relative folds of Tat-induced apoptosis normalized to the untreated ubiquitin-K0 mutant transfection group. Experiments were done in duplicate, 80 GFP-positive cells per group were counted, and the results were expressed as the mean ± SD. Two-tailed Student’s *t*-test for all graphs. *P < 0.05, **P <0.01, ***P < 0.001; ns, not significant.

By fluorescence microscopic analysis of cell morphology of GFP-positive cells, we found that cotransfection with ubiquitin-K29 and -K63 led to about 40% increase of cell shrinkage and cell membrane shriveling [24] when compared to the untreated ubiquitin-K0 cotransfection group (Figure 4A and B). The addition of MG132 led to about 40% increase of apoptosis in ubiquitin-K48 cotransfection groups, but caused no notable changes in other groups (Figure 4A and B). By Annexin V-APC staining coupled with flow cytometry, we found that cotransfection with ubiquitin-K29 caused a more than 2-fold increase of Tat-induced apoptosis than the other four groups in the absence of MG132 (Figure 4C and D). The addition of MG132 led to slight changes of Tat-induced apoptosis in ubiquitin-K0 or ubiquitin-K29 cotransfection groups, moderate increases in ubiquitin-K48 or ubiquitin-K63 cotransfection groups, and significant increases in ubiquitin-WT cotransfection groups (Figure 4C and D). The slight differences between the results of the two apoptosis analysis assays may be caused by manual counting in the former assay, which relies on subjective judgments to a certain extent.

**Figure 5:**
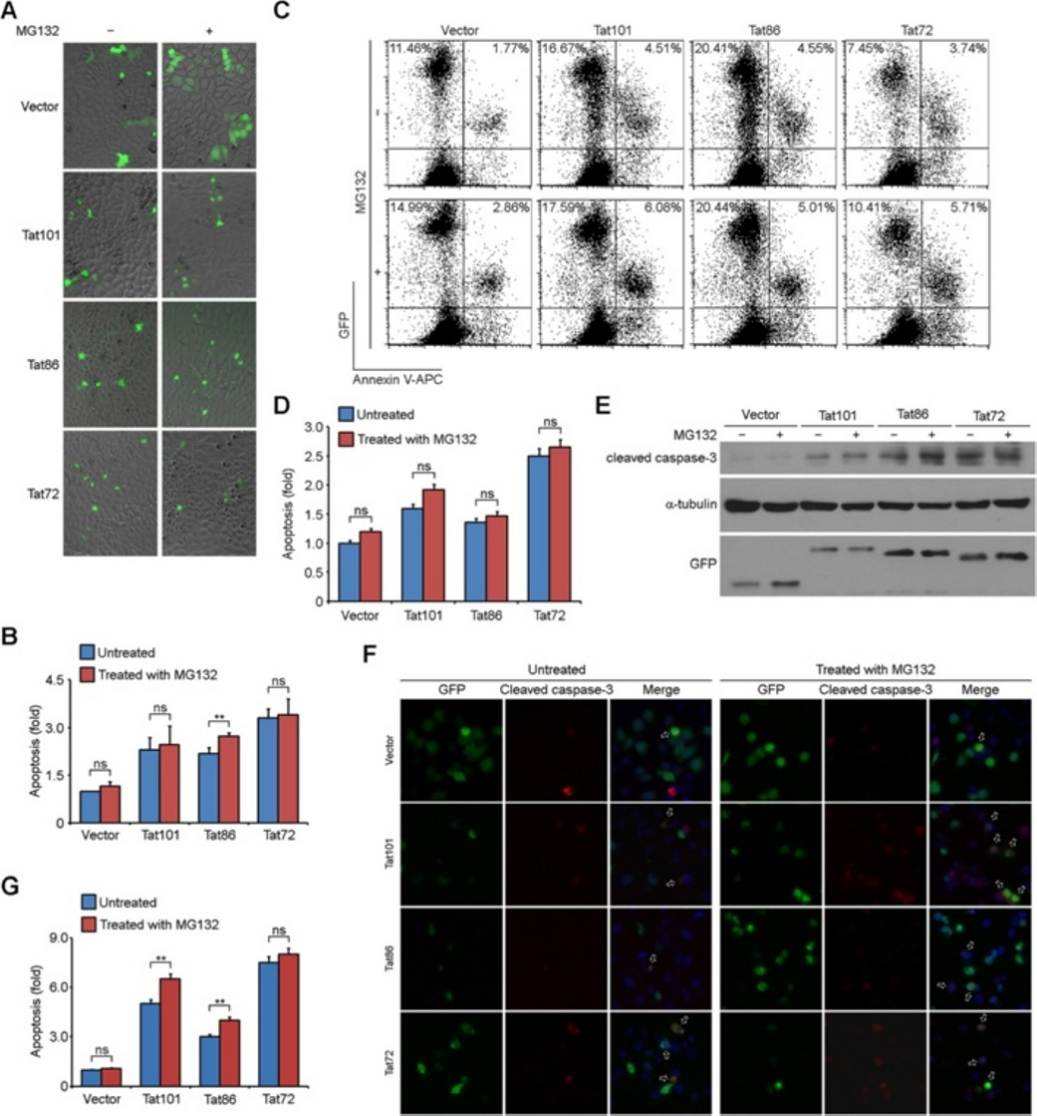
**Tat-induced apoptosis is modulated by its carboxyl-terminal region. (A)** HeLa cells were transfected with GFP-Tat101, GFP-Tat86, GFP-Tat72 or GFP alone. 72 hours post-transfection, cells were treated with (+) or without (-) MG132 and incubated for additional 24 hours. Apoptotic cells were examined via the fluorescence microscope by analyzing cell morphology of GFP-positive cells. **(B)** Quantification of the results in **(A)**. Experiments were done in duplicate, six fields per group were counted, and the results were expressed as the mean ± SD. **(C)** HeLa cells were transfected and treated as in **(A)**, and the apoptotic cells were examined by Annexin V-APC staining coupled with flow cytometry. **(D)** Quantification of the results in **(C)**. Bars represent the relative folds of Tat-induced apoptosis normalized to the untreated GFP vector transfection group. Mean and standard deviations were derived from two independent experiments done in duplicate. **(E)** HeLa cells were transfected and treated as in **(A)** and collected 96 hours post-transfection. Cell lysates were subjected to immunoblot analysis with antibodies specific for cleaved caspase-3, α-tubulin, or GFP, respectively. **(F)** HeLa cells were transfected and treated as in **(A)**, and stained with anti-cleaved caspase-3 antibodies and the DNA dye DAPI. Apoptotic cells were indicated by hollow arrows. **(G)** Quantification of the results in **(F)**. Bars represent the relative folds of Tat-induced apoptosis normalized to the untreated GFP vector transfection group. Experiments were done in duplicate, 80 GFP-positive cells per group were counted, and the results were expressed as the mean ± SD. Two-tailed Student’s *t*-test for all graphs. *P < 0.05, **P <0.01, ***P < 0.001; ns, not significant. Cropped blots are used in this figure, and the gels were run under the same experimental conditions.

Given that activation of caspase-3 is involved in the process of Tat-induced apoptosis [25], we then assessed the percentage of cleaved caspase-3 (the active form of caspase-3) positive cells 96 hours post-transfection. As shown in Figure 4E, HeLa cells with overexpressed GFP-Tat101, which were positive for cleaved caspase-3, were indicated by hollow arrows. The percentage of cleaved caspase-3 positive cells in ubiquitin-K29 cotransfection group was higher than that of the other four groups in the absence or presence of MG132 respectively (Figure 4F), which concurred with the results in Figure 4B and D. Cotransfection with ubiquitin-WT, ubiquitin-K48, or ubiquitin-K63 led to an increase in Tat-induced apoptosis when compared to the ubiquitin-K0 cotransfection groups (Figure 4F), which concurred with the data in Figure 4D.With similar approaches, we assessed the effects of the carboxyl-terminal region of Tat on its ability to induce apoptosis. By fluorescence microscopy and flow cytometry, we found that Tat72 was the most potent variant of Tat to trigger apoptosis (Figure 5A-D). The induction of apoptosis by the three forms of Tat was scarcely affected by MG132 treatment (Figure 5D). Immunoblot analysis and immunofluorescence microscopy also showed that GFP-Tat72 overexpression caused the most robust activation of caspase-3 (Figure 5E-G). Thus, these results indicate that Tat-induced apoptosis is regulated by its ubiquitination and carboxyl-terminal region.

### The ubiquitination and carboxyl-terminal region of Tat influence its activity towards microtubule assembly

**Figure 6:**
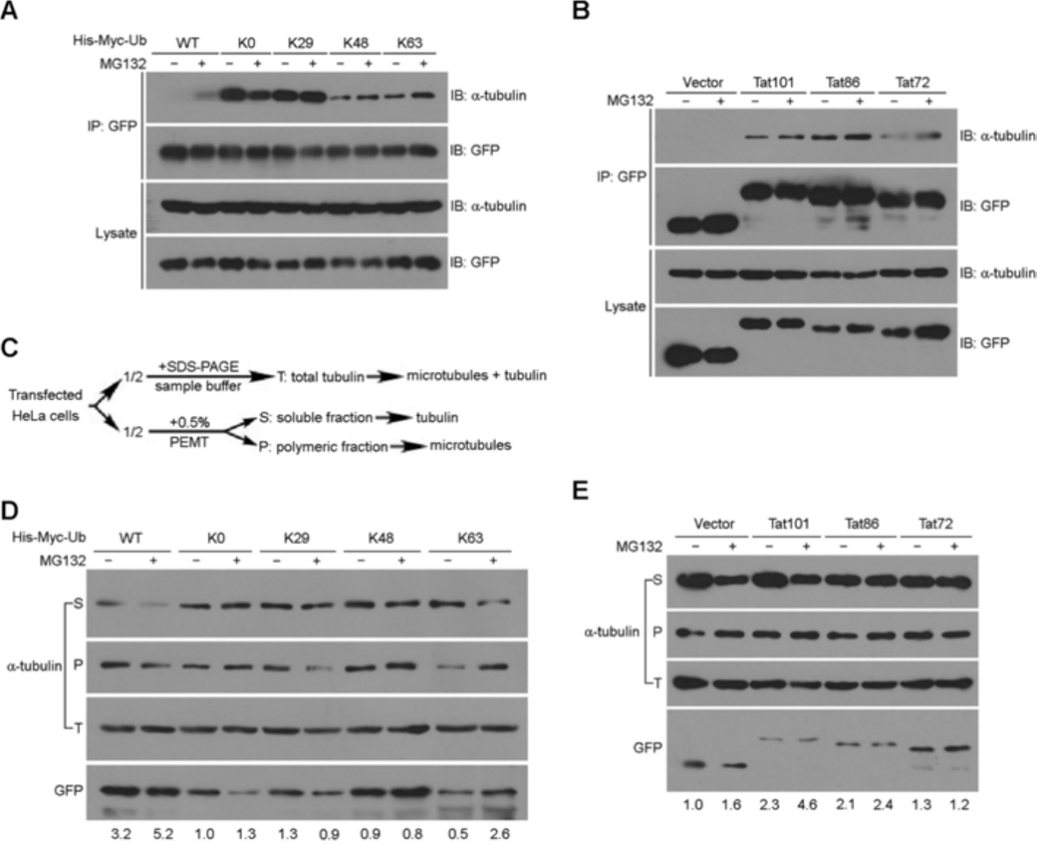
**The ubiquitination and carboxyl-terminal region of Tat influence its activity towards microtubule assembly. (A)** Anti-GFP immunoprecipitates and cell lysates were immunoblotted with anti-α-tubulin or anti-GFP antibodies. **(B)** 293 T cells were transfected with GFP-Tat101, GFP-Tat86, GFP-Tat72 or GFP alone and treated with (+) or without (-) MG132. Anti-GFP immunoprecipitates and cell lysates were subjected to immunoblot analysis with antibodies against α-tubulin or GFP. **(C)** Schematic representation of the experimental workflow. Upon transfection of HeLa cells with the indicated plasmids, half the cells were boiled in SDS-PAGE sample buffer to obtain a mixture of soluble tubulin and polymeric fraction (microtubules) as total tubulin (T). The other half were subjected to the preparation of soluble tubulin (S) and polymeric fraction (P) as described in Materials and Methods. **(D)** HeLa cells were cotransfected with GFP-Tat101 and various ubiquitin mutants and treated with (+) or without (-) MG132. Protein samples were prepared as in **(C)** and immunoblotted with antibodies against α-tubulin or GFP. The ratios of polymeric fraction over soluble tubulin (P/S) were normalized against total tubulin levels and GFP intensity, and the relative folds were normalized to the untreated ubiquitin-K0 mutant transfection group and shown under the corresponding blots. **(E)** HeLa cells were transfected with GFP-Tat101, GFP-Tat86, GFP-Tat72 or GFP alone and treated with (+) or without (-) MG132. Protein samples were prepared as in **(C)** and analyzed as in **(D)**. Cropped blots are used in this figure, and the gels were run under the same experimental conditions.

Previous studies have elucidated that Tat directly interacts with tubulin dimers and microtubules, which alters the microtubule dynamics and contributes to Tat-induced apoptosis [23, 25–29].We hypothesized that the ubiquitination and carboxyl-terminal region of Tat might regulate Tat-induced apoptosis by modulating its activity towards microtubule assembly.To test this hypothesis, we firstly examined the interaction between Tat and tubulin in response to the ubiquitination or carboxyl-terminal region truncation of Tat. By immunoprecipitation assays, we found that cotransfection with ubiquitin-K48 or -K63 mutants dramatically diminished the interaction of Tat with tubulin as compared to the K0 group (Figure 6A). MG132 treatment remarkably enhanced the Tat-tubulin interaction in WT group, but only slightly increased their interaction in ubiquitin-K48 or -K63 groups (Figure 6A). Surprisingly, among the three variants of Tat, Tat86 displayed the strongest interaction with tubulin (Figure 6B). Only the interaction between Tat72 and tubulin was notably enhanced by the treatment of MG132 (Figure 6B).We then investigated the effects of the ubiquitination and carboxyl-terminal region of Tat on its activity towards microtubule assembly. Protein samples containing soluble tubulin (S), polymerized microtubules (P) or a mixture of both (T) were prepared from HeLa cells transfected with the indicated plasmids (Figure 6C) and analyzed by immunoblot. Compared with the K0 group, cotransfection with ubiquitin-WT elevated Tat ability to stabilize microtubules, which was further promoted by the addition of MG132 (Figure 6D). Curiously, the effect of ubiquitin-K63 on Tat stabilization of microtubules was sharply changed by MG132 treatment, from inhibition to promotion (Figure 6D). In addition, the two truncated forms of Tat showed weaker microtubule-stabilizing abilities with limited response to MG132 treatment (Figure 6E). Nevertheless, a two-fold increase in microtubule-stabilizing activity of full-length Tat by MG132 treatment was observed in this set of experiments (Figure 6E). Taken together, these data demonstrate that the activity of Tat towards microtubule assembly is affected by its ubiquitination and carboxyl-terminal region.

## Discussion

Emerging evidence suggests that posttranslational modifications play critical roles in regulating various protein functions. The consequences can be the alteration of protein structure, stability, subcellular localization, or even protein activities. The Tat protein of HIV-1 has been demonstrated to be modulated by a variety of posttranslational modifications [22]. Prior to our study, Tat has been reported to undergo lysine 63-linked ubiquitination that does not affect Tat stability [2]. However, according to our results, Tat is modified by lysine 48-linked polyubiquitin chains and targeted to proteasome-dependent degradation. The divergence is probably due to the fact that the plasmids encoding ubiquitin mutants used in the two studies are different. That is, the ubiquitin mutants used in their study contain a single lysine mutation, while ours maintain only one lysine with all the others mutated to arginine. Multipoint mutation may affect the occurrence of certain types of ubiquitination. Although the lysine 29- or 63-linked ubiquitination of Tat failed to be detected, cotransfection with one of the two mutants definitely affected Tat activities. Nevertheless, we could not rule out the possibility that overexpression of ubiquitin could influence the interaction partners of Tat, which indirectly contributes to the regulatory effects on Tat.

Our study also shows that the amount of Tat72 was increased after MG132 treatment, whereas Tat86 showed little response. The sequences of these two truncated forms are identical apart from the presence of a RGD motif at the carboxyl-terminal region of Tat86, whose well-characterized role is to enable Tat protein to bind cell membranes [30]. Although there is no evidence of a correlation between the RGD motif and proteasome-dependent degradation of Tat proteins, the difference of the two truncated proteins in response to MG132 treatment is as a result of the additional RGD motif of Tat86. The protein structure may be changed by the addition of the RGD motif, which may further interfere the interaction between Tat86 and its E3 ubiquitin ligases and affect the protein stability.

In our study, although the subcellular localization of Tat was not influenced by coexpression with various ubiquitin mutants, the transactivation activity was affected to varying degrees. A previous study has reported that lysine 63-linked ubiquitination of Tat increases its transactivation activity [2]. We did not observe that type of ubiquitination on Tat, but cotransfection with ubiquitin-K63 mutant did promote Tat-mediated transactivation. Lysine 48-linked ubiquitination of Tat remarkably enhanced its transactivation activity in the presence of the proteasome inhibitor MG132, which indicates that the ubiquitin-proteasome system is involved in the regulation of Tat-mediated transactivation. In the ubiquitin-WT cotransfection groups, the changes of Tat transactivation activity caused by MG132 treatment were not significant, which might be due to competitive inhibition of lysine 48-linked ubiquitination and proteasome-dependent degradation of Tat by the other lysines in wild-type ubiquitin.

Additionally, the transactivation activity of Tat appears to be exquisitely regulated by its carboxyl-terminal region. Our data reveal that the transactivation activity of full-length Tat is higher than that of Tat86, which concurs with previous reports [19], but lower than that of Tat72. Only the transactivation activity of Tat72 was strikingly enhanced by the addition of MG132.

Besides the well-known transactivation activity, Tat also exerts a variety of other biological activities such as induction of apoptosis. According to the previously published studies, Tat-induced apoptosis does not correlate with its transactivation activity towards the HIV-1 LTR [19]. In agreement with these findings, our results show that there is no apparent correlation between these two functions of the three forms of Tat or when the full-length Tat was cotransfected with various ubiquitin plasmids.

Several studies have reported that Tat promotes the killing of cells by targeting the microtubule network [23, 25–29]. Tat can directly interact with tubulin dimers and polymerized microtubules, which results in the abnormal stabilization of microtubules and the disturbance of microtubule dynamics [26, 28]. It has been shown previously that the four residues (36–39) of Tat are necessary and sufficient for its interaction with tubulin [26]. We additionally demonstrate that the carboxyl-terminal region of Tat does have some impact on this interaction. However, Tat-induced apoptosis does not entirely result from its microtubule-stabilizing abilities. Among the three forms of Tat, Tat72, the form generated in the late stage of HIV-1 infection cycle, has the weakest activity towards microtubule assembly but induces the most robust apoptosis, which may promote the progression of infection and lead to pathogenesis of the acquired immunodeficiency syndrome (AIDS). Coexpression with various ubiquitin mutants definitely affects Tat-induced apoptosis and its activity towards microtubule assembly, but there is no apparent correlation between the two aspects.

## Conclusions

Tat, a multidomain and multifunction protein, plays vital roles in HIV-1 replication cycle and the pathogenesis of AIDS [17, 31, 32]. The present study reveals that truncation of the carboxyl-terminal region of Tat leads to functional differences. Thus, it is important to give sufficient consideration to the carboxyl-terminal region of Tat in future researches. As reported previously, a large number of transcription factors are regulated by ubiquitin-proteasome system [33]. Our study adds Tat to the list. The stability and activities of Tat are modulated by ubiquitination. Although a variety of posttranslational modifications have been reported to occur on Tat [22], their exact effects are far from fully characterization. Targeting Tat posttranslational modifications may represent a new and unique therapy for HIV-1 sufferers. As evidenced by publications, Tat acetylation is a dynamic and highly regulated process during HIV-1 infection [4, 9, 10, 12, 14]. Locking Tat in one modified state may diminish or even abolish Tat activities which are essential for HIV-1 infection and AIDS progression. Maybe this strategy can be applied to other types of Tat posttranslational modifications that will facilitate AIDS treatment.

## Materials and methods

### Antibodies and plasmid constructs

Antibodies against GFP (Roche), Myc (Sigma-Aldrich), α-tubulin (Abcam), and cleaved caspase-3 (Cell Signaling Technology), horseradish peroxidase-conjugated secondary antibodies (Amersham Biosciences), and rhodamine-conjugated secondary antibodies (Jackson ImmunoResearch Laboratories) were obtained from the indicated sources. The mammalian expression plasmid for GFP-Tat101 was constructed by cloning HIV-1 Tat cDNA into the pEGFPC1 vector, and the truncated forms, GFP-Tat86 and GFP-Tat72, were generated by insertion of the cDNA fragments into the pEGFPC1 vector. The His-Myc-ubiquitin expression plasmid was kindly provided by Ceshi Chen (Kunming Institute of Zoology, Chinese Academy of Sciences), and the K0, K29, K48, and K63 mutants were generated by site-directed mutagenesis. The ubiquitin-K0 mutant contains no lysine, and the ubiquitin-K29, -K48, and -K63 mutants contain only a single lysine (K29, K48, and K63, respectively), with all the other lysines mutated to arginine. The HIV-1 long terminal repeat (LTR)-driven luciferase plasmid has been described previously [14]. All plasmids were verified by DNA sequencing.

### Cell transfection and treatment

293 T cells and HeLa cells were obtained from the American Type Culture Collection. Plasmids were transfected to 293 T cells by using the polyethyleneimine reagent (Polysciences) and transfected to HeLa cells by using Entranster™-D (Engreen Biosystem). For the inhibition of proteasome, 5 μM MG132 (Sigma-Aldrich) was added into the culture medium and cells were incubated for additional 8 hours unless indicated otherwise.

### Quantitative real-time PCR analysis

293 T cells transfected with GFP-Tat101, GFP-Tat86, or GFP-Tat72 were treated with (+) or without (-) MG132. Total RNA was isolated from the cells using the TRIzol reagent (Invitrogen) according to the manufacturer’s instruction. The isolated RNA was reverse-transcribed into cDNA by reverse transcriptase (Promega). The following primer sequences (listed later) were used. Quantitative real-time PCR reactions were performed using SYBR green master mix (BioRad) with an Eppendorf realplex. Primers used were as follows. *Tat*-forward, GTTTGTTTCACAAAAAAAGGCTTAG, *Tat*-reverse, CTAGTTTAGGATCTACTGGCTCCAT; human *GAPDH*-forward, ATCACTGCCACCCAGAAGAC, human *GAPDH*-reverse, ATGAGGTCCACCACCCTGTT.

### Preparation of soluble and polymeric tubulin

HeLa cells were lysed in the PEMT buffer (100 mM PIPES, 1 mM EGTA, 1 mM MgSO_4_, 1 mM GTP, 4 M glycerol, 0.5% Triton X-100, pH 6.8), and the supernatant was then collected as soluble tubulin. The remaining polymeric fraction was dissolved in 2% SDS in 50 mM Tris, pH 6.8.

### Immunoblot analysis and immunoprecipitation

Protein samples were resolved by SDS-PAGE and transferred onto polyvinylidene difluoride membranes (Millipore). The membranes were blocked with 5% fat-free milk in Tris-buffered saline containing 0.1% Tween 20 and incubated with primary antibodies followed by horseradish peroxidase-conjugated secondary antibodies. Specific proteins were visualized with enhanced chemiluminescence detection reagent following the manufacturer’s instructions (Pierce Biotechnology). For immunoprecipitation, protein samples were incubated with anti-GFP antibody-conjugated agarose beads (MBL) at 4°C overnight. The beads were washed extensively and subjected to immunoblot analysis.

### Immunofluorescence microscopy

HeLa cells grown on glass coverslips were fixed with 4% paraformaldehyde for 30 minutes at room temperature followed by permeabilization with 0.5% Triton X-100 in PBS for 20 minutes. Cells were then blocked with 2% bovine serum albumin in PBS for 30 minutes and incubated in succession with the primary antibody and rhodamine-conjugated secondary antibody followed by staining with DAPI (Sigma-Aldrich) for 3 minutes. Coverslips were mounted with 90% glycerol in PBS and images were obtained using an Axio Observer A1 fluorescence microscope (Carl Zeiss Inc).

### Luciferase reporter assay

Plasmids to be detected were cotransfected with the HIV-1 LTR-driven luciferase plasmid to 293 T cells. 24 hours post-transfection, the fluorescence of GFP was examined by using the fluorescence microscope, and the intensity of GFP-tagged proteins was assessed via ImageJ software. Cells were then lysed and the transactivation activity was measured with an FB12 luminometer (Berthold Detection Systems) and normalized to GFP intensity.

### Apoptosis analysis

HeLa cells were examined under the fluorescence microscope 96 hours post-transfection, and both phase contrast and fluorescence images were taken. The percentage of apoptotic cells was quantified by analyzing cell morphology of the GFP-positive cells. Additionally, apoptotic cells were also examined and quantified by Annexin V-APC (BioLegend) staining coupled with flow cytometry.

### Statistical analysis

Analysis of statistical significance was performed by two-tailed Student’s *t*-test using GraphPad Prism 5. *P < 0.05, **P <0.01, ***P < 0.001; ns, not significant. Data represent means ± SD.
